# Diagnostic value of ileocolic artery and vein diameters in acute appendicitis: A retrospective observational study with contrast-enhanced computed tomography

**DOI:** 10.1097/MD.0000000000050277

**Published:** 2026-08-21

**Authors:** Davut Unsal Capkan

**Affiliations:** a Department of Radiology, Private Medical Point Hospital, Gaziantep, Turkey.

**Keywords:** acute appendicitis, computed tomography, diagnostic accuracy, ileocolic artery, ileocolic vein

## Abstract

Acute appendicitis is a common surgical emergency characterized by right lower quadrant pain. This study investigated the diagnostic value of ileocolic artery (ICA) and ileocolic vein (ICV) diameters measured using contrast-enhanced computed tomography (CT) in patients with suspected acute appendicitis. This retrospective observational study included 203 patients aged 18 to 70 years who underwent CT for abdominal pain between January 2022 and December 2024, comprising 97 patients with acute appendicitis and 106 controls. ICA and ICV diameters were measured within the proximal 3 cm segments arising from the superior mesenteric vessels. Data were analyzed using the Mann–Whitney *U* test, receiver operating characteristic analysis, and multivariable logistic regression. The appendicitis group had significantly larger ICA and ICV diameters than the control group (ICA: 2.86 ± 0.43 mm vs 2.03 ± 0.48 mm; ICV: 4.56 ± 0.72 mm vs 3.58 ± 0.66 mm; both *P* < .001). ICA diameter showed an area under the curve of 0.915 at a cut-off value of 2.25 mm, with 95.9% sensitivity and 75.5% specificity, whereas ICV diameter showed an area under the curve of 0.854 at a cut-off value of 4.05 mm, with 81.4% sensitivity and 77.4% specificity. Multivariable logistic regression identified both ICA and ICV diameters as independent predictors of acute appendicitis (ICA: odds ratio = 26.78, 95% confidence interval: 9.91–86.72; ICV: odds ratio = 8.38, 95% confidence interval: 3.83–20.63). ICA and ICV diameter measurements may provide objective and quantitative adjunctive markers for diagnosing acute appendicitis but should be interpreted alongside conventional CT findings and clinical evaluation.

## 1. Introduction

Acute appendicitis constitutes a major cause of urgent surgical intervention globally, with diagnostic delays potentially resulting in increased morbidity. Typically resulting from obstruction of the appendiceal lumen, it triggers a cascade of inflammatory responses, including changes in vascular structures. While clinical examination, laboratory testing, and radiologic imaging are commonly employed together, diagnosis may still be challenging in atypical presentations.^[1–3]^

Owing to its excellent sensitivity and specificity, computed tomography (CT) has become the standard approach in the diagnostic evaluation of suspected acute appendicitis. Classic CT findings include appendiceal dilation, wall thickening, increased enhancement, and periappendiceal fat stranding. However, increasing attention has been given to the vascular implications of inflammation, such as local vasodilatation and hyperemia, which may be reflected in the diameter changes of surrounding vessels.^[4,5]^

The appendiceal artery typically originates as a terminal offshoot of the ileocolic artery (ICA), while venous drainage of the appendix is primarily facilitated by the ileocolic vein (ICV). It is hypothesized that in acute inflammation, the diameters of these vessels may increase as a response to the local hyperemic state. Yet, the literature hosts a paucity of research exploring the link between ICA and ICV diameters and acute appendicitis. Thus, this study seeks to contribute to clinical practice by quantitatively analyzing ICA and ICV diameters using contrast-enhanced CT.^[6,7]^

Accordingly, the present study aimed to evaluate whether the diameters of the ICA and vein measured on contrast-enhanced CT are significantly increased in patients with acute appendicitis compared with controls, and to assess their potential role as independent diagnostic predictors.

## 2. Method

This study was approved by the Ethics Committee of Gaziantep City Hospital (Protocol No: E-227553161-514.10-235233430; Approval No: 142/2025, Date: March 19, 2025). Given the retrospective design, the requirement for informed consent was waived by the ethics committee. All procedures were conducted in accordance with the principles of the Declaration of Helsinki. We retrospectively evaluated the data from individuals aged 18 to 70 years who presented with abdominal pain and underwent contrast-enhanced abdominal CT at our emergency department between January 2022 and December 2024. We included those with portal venous phase images and sufficient clinical and radiological data. Nevertheless, patients with known hematologic, oncologic, vascular, or endothelial disorders, incomplete data, or pregnancy were excluded.

**Figure 1.**
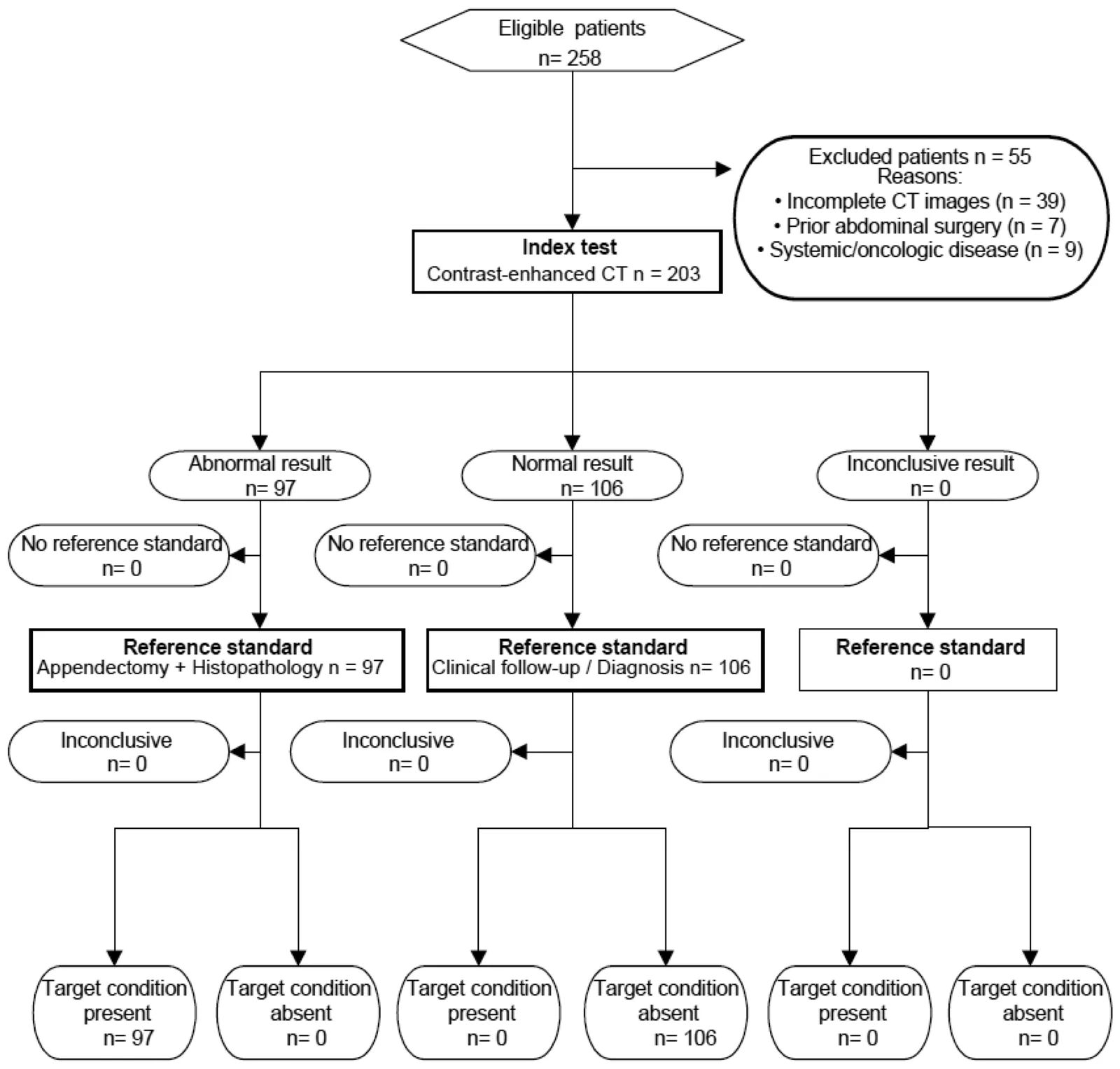
STARD flow diagram of patient selection and diagnostic accuracy analysis.

A total of 258 patients who underwent abdominal CT for abdominal pain between January 2022 and December 2024 were initially screened for eligibility. After applying exclusion criteria including incomplete clinical or imaging data and known systemic disease, 55 patients were excluded. The remaining 203 patients met the inclusion criteria. Of these, 97 patients with CT findings consistent with acute appendicitis who subsequently underwent appendectomy and had surgical and/or histopathological confirmation were assigned to the appendicitis group. The remaining 106 patients without radiographic evidence of appendicitis formed the control group (Figure 1). The control group included patients who underwent abdominal CT for right lower quadrant or diffuse abdominal pain but had no radiological evidence of appendicitis. The most common alternative diagnoses in this group were nonspecific abdominal pain, gastroenteritis, mesenteric lymphadenitis, ovarian cysts, and urinary tract infection.

Abdominal CT was conducted using a 256-detector row CT scanner, encompassing the anatomical region between the hepatic dome and the pelvic base. Image acquisition parameters included a tube voltage range of 120 to 140 kVp and a section thickness of 5 mm. Intravenous administration of a nonionic iodinated contrast medium was carried out at a rate of 2 to 4 mL per second, with portal venous phase images obtained approximately 1-minute post injection.

**Figure 2.**
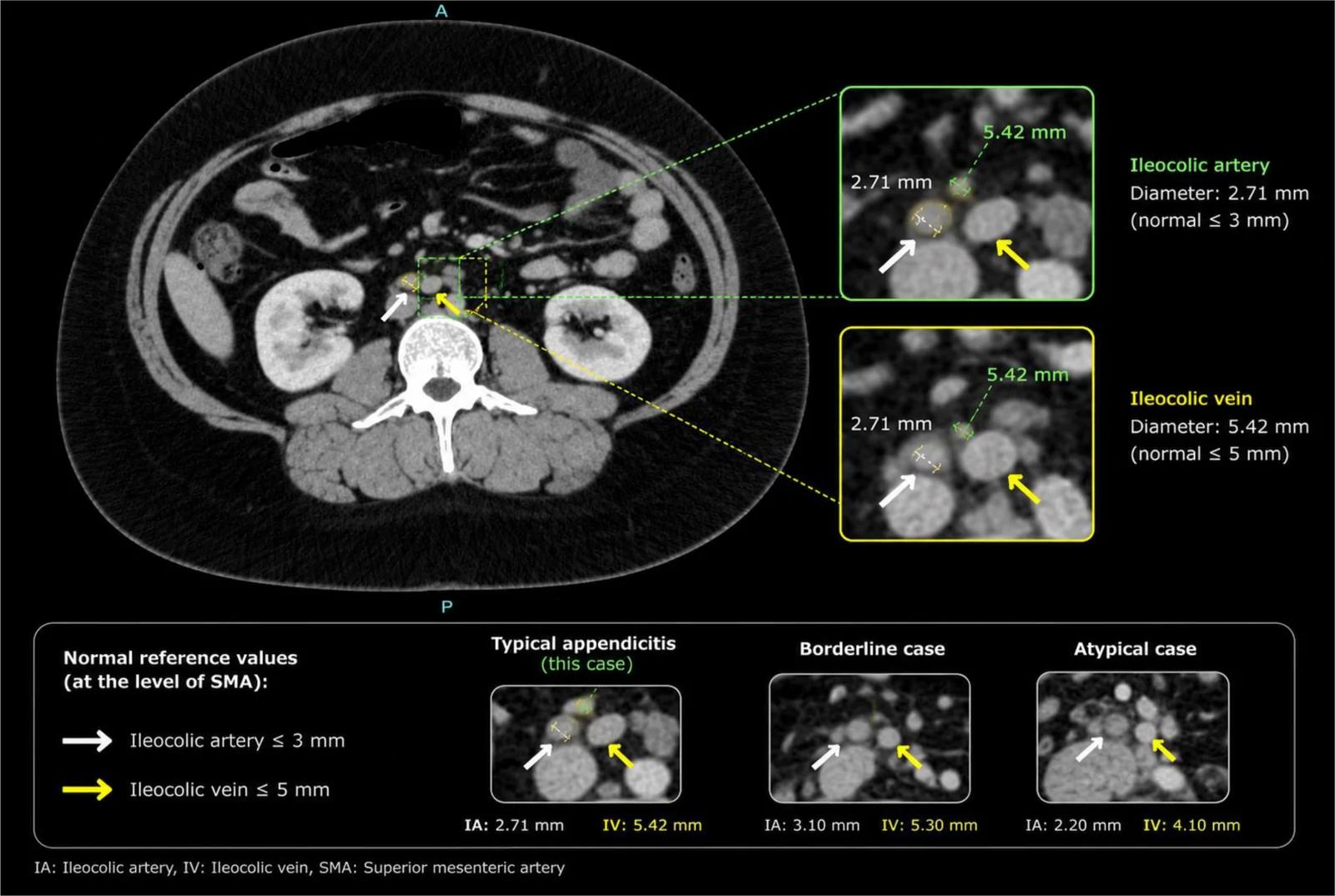
Axial contrast-enhanced abdominal CT image at the level of the superior mesenteric vessels in a patient with acute appendicitis, measurement of the ileocolic artery (white arrow) and ileocolic vein (yellow arrow). CT = computed tomography.

An experienced radiologist independently carried out all measurements using digital calipers on axial CT images. The radiologist performing ICA and ICV diameter measurements was blinded to the final surgical and histopathological diagnosis during image assessment. ICA and ICV diameters were measured within the proximal 3 cm segments originating from the superior mesenteric vessels (Figure 2).

The data are summarized as mean ± standard deviation. We analyzed the data using SPSS 21.0. The Mann–Whitney *U* test was employed to compare non-normally distributed data. Receiver operating characteristic analysis was performed to establish cutoff values and assess diagnostic performance in terms of sensitivity and specificity. The variables were modeled through multivariate logistic regression to determine independent diagnostic indicators. No formal a priori sample size calculation was performed because of the retrospective design. The final sample size was determined by the number of eligible patients who met the inclusion criteria during the predefined study period. A *P*-value < .05 was considered significant in all analyses.

Patients with incomplete clinical or imaging data were excluded from the study. No imputation methods were used, and all analyses were performed with complete case data.

## 3. Results

While 97 (47.8%) patients fell into the appendicitis group, 106 (52.2%) served as controls. Within the control group, the most frequent clinical diagnoses were nonspecific abdominal pain (n = 38), gastroenteritis (n = 22), mesenteric lymphadenitis (n = 18), gynecological conditions such as ovarian cysts (n = 16), and urinary tract infection (n = 12). Patients’ gender distribution was nearly overlapping (males = 51.2% (n = 104), females = 48.8% (n = 99)). The mean age was computed to be 35.98 ± 11.76 years, and the mean ICA and ICV diameters were 2.43 ± 0.62 mm and 4.05 ± 0.84 mm, respectively.

**Figure 3.**
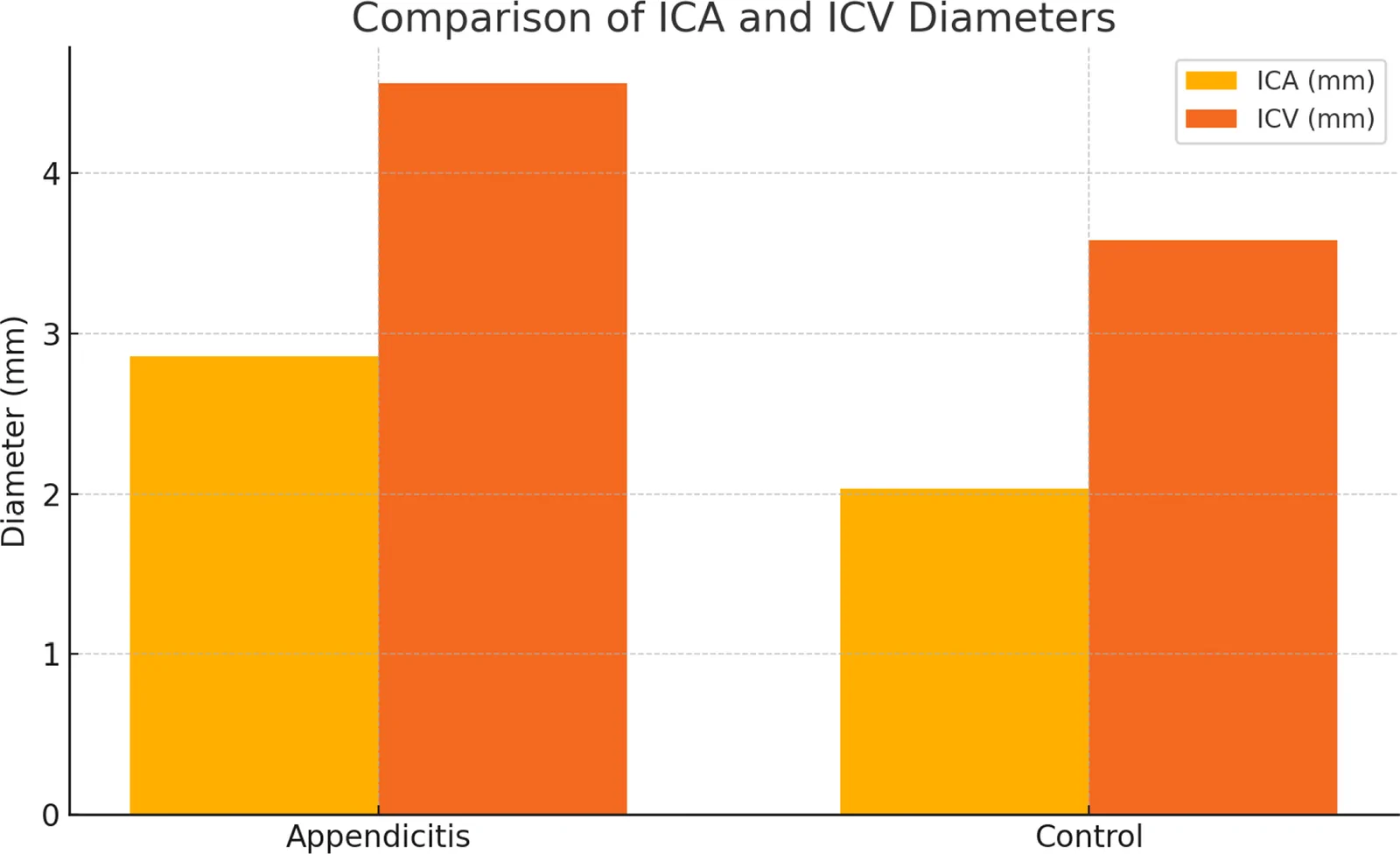
Comparison of ileocolic artery (ICA) and vein (ICV) diameters between appendicitis and control groups. ICA indicates ileocolic artery; ICV, ileocolic vein. Values are presented as mean ± standard deviation.

The appendicitis group demonstrated significantly larger ICA and ICV diameters compared with controls (ICA: 2.86 ± 0.43 mm vs 2.03 ± 0.48 mm; ICV: 4.56 ± 0.72 mm vs 3.58 ± 0.66 mm; both *P* < .001). In contrast, there were no significant differences between the groups with respect to age or sex (*P* > .05) (Figure 3).

**Figure 4.**
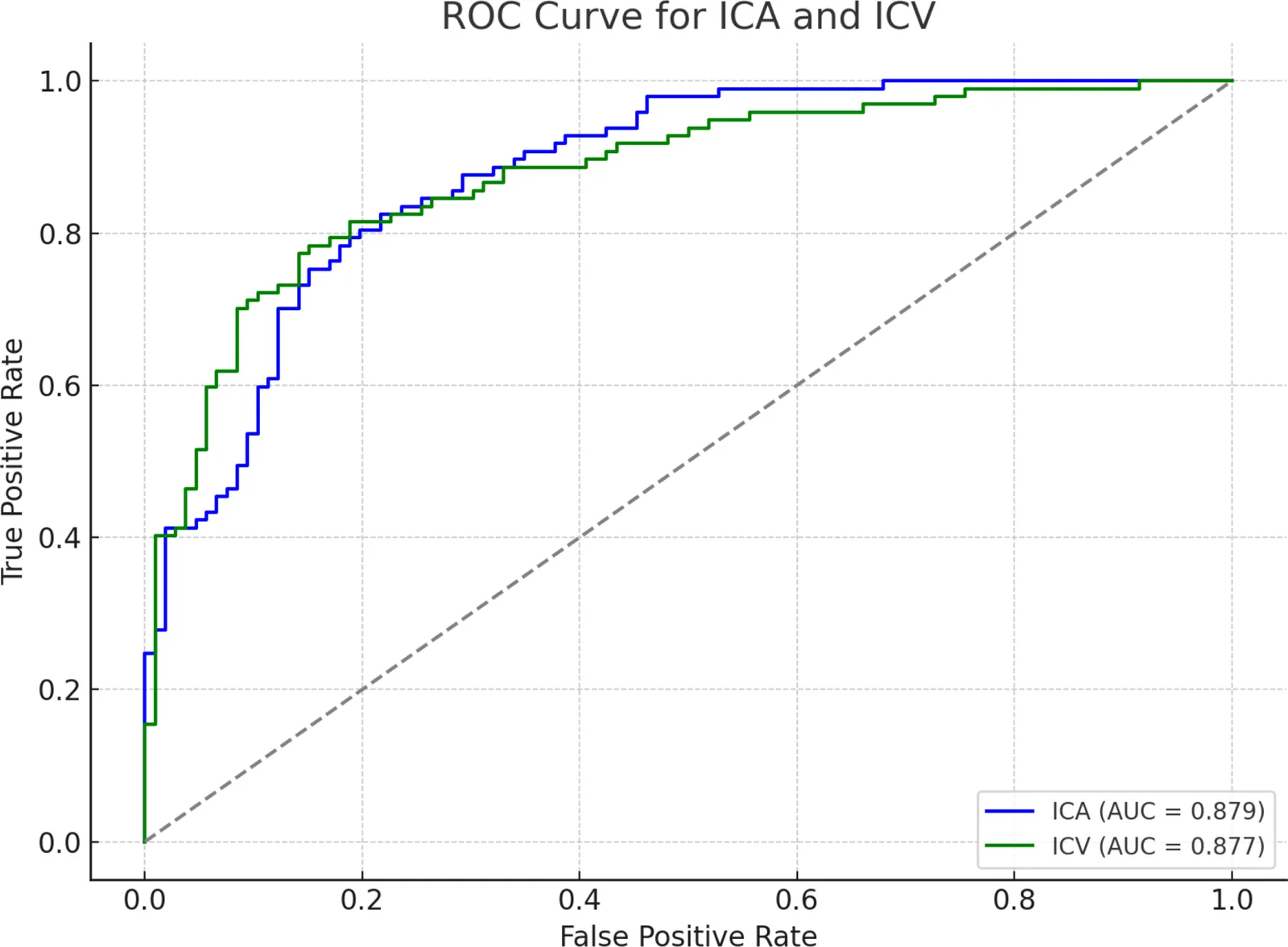
Logistic regression for ICA and ICV diameters. ICA and ICV diameters were identified as independent predictors of acute appendicitis. Shown are odds ratios (OR) with 95% confidence intervals. CI = confidence interval, ICA = ileocolic artery, ICV = ileocolic vein, ROC = receiver operating characteristic.

Multivariate logistic regression analysis identified both ICA and ICV diameters as independent predictors of acute appendicitis (ICA: odds ratio (OR) = 26.78, 95% confidence interval (CI): 9.91–86.72, *P* < .001; ICV: OR = 8.38, 95% CI: 3.83–20.63, *P* < .001) (Figure 4).

receiver operating characteristic curve analysis further demonstrated high diagnostic performance. For ICA diameter, the area under the curve (AUC) was 0.915 (95% CI: 0.873–0.957), with a cutoff value of 2.25 mm yielding a sensitivity of 95.9% and specificity of 75.5%. For ICV diameter, the AUC was 0.854 (95% CI: 0.802–0.907), with a cutoff value of 4.05 mm corresponding to a sensitivity of 81.4% and specificity of 77.4%.

## 4. Discussion

This study demonstrated that ICA and ICV diameters measured on contrast-enhanced CT are significantly increased in patients with acute appendicitis and can serve as independent diagnostic predictors. These findings highlight the role of vascular changes accompanying inflammation and expand the diagnostic framework beyond conventional CT signs such as appendiceal dilation, wall thickening, and periappendiceal fat stranding.

Our results are in line with the growing body of literature emphasizing vascular alterations in acute appendicitis. Sirik et al reported that both ICA and ICV diameters were significantly larger in patients with acute appendicitis compared with controls, supporting the notion that local hyperemia and vasodilatation are measurable radiological markers.^[8]^ Kuzan et al demonstrated that ileocolic vessel diameters could be utilized as novel parameters in the diagnosis of appendicitis, although the absolute values they reported were higher than those in our cohort. This discrepancy may reflect differences in patient demographics, imaging protocols, or measurement methodologies, but the direction of association remains consistent.^[9]^

Barac et al also explored ileocolic vessel diameters and found that body mass index influenced vascular measurements, highlighting the importance of controlling for confounders in vascular imaging studies.^[7]^ Our results complement these findings by demonstrating strong diagnostic accuracy despite not adjusting for BMI or comorbidities, suggesting that ICA and ICV diameters may retain predictive value even in heterogeneous clinical populations.

In addition, Kartal et al recently confirmed the diagnostic accuracy of ICA and ICV diameters for acute appendicitis in a large patient sample, reporting high sensitivity and specificity values.^[6]^ Our findings mirror theirs, particularly regarding ICA, which emerged as the most powerful independent predictor with an AUC exceeding 0.90. Taken together, these studies underscore the reproducibility of vascular-based parameters across different populations and study designs.

Beyond vascular imaging, numerous investigators have assessed serum and inflammatory biomarkers to improve appendicitis diagnosis. For instance, Arredondo Montero et al demonstrated the utility of calprotectin, APPY-1, and CA-125 in distinguishing complicated from uncomplicated appendicitis, while Bueso-Inchausti Garcia et al highlighted copeptin as a promising biomarker in pediatric populations.^[10–13]^ Compared with these serum-based approaches, imaging-derived vascular measurements such as ICA and ICV diameters provide direct anatomical correlates of local inflammation and can be readily integrated into routine CT evaluation without additional laboratory testing.

Furthermore, Martin et al described inflammatory appendiceal masses and emphasized the diagnostic relevance of vascular involvement in CT imaging.^[14]^ Our findings support this concept by quantifying vascular changes that accompany inflammation, thereby offering a reproducible method that complements both structural CT findings and clinical scoring systems.

Overall, the present study contributes to the literature by validating ileocolic vessel diameters as reliable diagnostic markers in acute appendicitis. The high sensitivity observed for ICA measurements suggests a strong role in ruling out disease when normal, whereas the moderate specificity indicates that these parameters should not be used in isolation. Instead, they should be interpreted in conjunction with conventional CT features and clinical findings to optimize diagnostic accuracy and reduce unnecessary surgical interventions.

## 5. Limitations

The relatively large sample size and the use of a standardized measurement protocol conducted by a single experienced radiologist, thereby minimizing observer bias, can be counted among the notable strengths of this study. Nevertheless, the retrospective design inherently introduces certain limitations. In particular, potential confounding variables such as body mass index, cardiovascular comorbidities, and hydration status, which may influence vascular diameters, were not controlled for in the analysis. Therefore, the observed associations should be interpreted with caution. Furthermore, we were unable to perform subgroup analyses to differentiate between uncomplicated and complicated appendicitis due to the retrospective design and limited subgroup sample sizes. As a result, the relationship between appendicitis severity and ileocolic vascular changes could not be fully evaluated. Future prospective studies with larger cohorts should investigate whether ICA and ICV diameters vary according to disease severity and complication status.

In addition, intra-observer and inter-observer reliability analyses were not performed. Although all measurements were conducted by a single experienced radiologist using a standardized protocol to minimize variability, the absence of reproducibility analyses such as ICC or Bland–Altman agreement testing may limit both the reproducibility and generalizability of our findings across different observers and institutions. Therefore, future prospective multicenter studies incorporating independent repeated measurements by multiple readers are needed to externally validate the diagnostic utility of ICA and ICV diameters. Moreover, assessing the diameters of these vessels, particularly in patients with atypical clinical presentations, may help reduce the rate of avoidable surgical interventions.

## 6. Conclusion

In conclusion, this study demonstrated that measuring ICA and ICV diameters using contrast-enhanced CT may serve as a valuable diagnostic tool in acute appendicitis. ICA and ICV diameters were significantly elevated in appendicitis patients, and these parameters showed high sensitivity and specificity in diagnosis. Threshold values of 2.25 mm for ICA and 4.05 mm for ICV may serve as objective and quantitative indicators in clinical decision-making processes. Thus, assessing ileocolic vessel diameters along with standard CT findings is likely to help improve the diagnostic accuracy in suspected cases of acute appendicitis. Given the high sensitivity but only moderate specificity of these parameters, their use in clinical practice should be complementary rather than standalone. ICA and ICV measurements may serve as supportive markers that can increase diagnostic confidence, particularly in patients with atypical presentations, but they should always be interpreted in conjunction with conventional CT findings and clinical evaluation to minimize false-positive results and avoid unnecessary surgical interventions.

## Acknowledgments

The author would like to express sincere gratitude to Prof Dr İbrahim Tayfun Şahiner, Department of General Surgery, for his valuable guidance and contributions during the course of this study. The author also thanks Mr. Fatih Usen for his support with the statistical part of the study. Both individuals named in the Acknowledgments have given their permission to be acknowledged in this publication.

## Author contributions

**Conceptualization:** Davut Unsal Capkan.

**Data curation:** Davut Unsal Capkan.

**Formal analysis:** Davut Unsal Capkan.

**Funding acquisition:** Davut Unsal Capkan.

**Investigation:** Davut Unsal Capkan.

**Methodology:** Davut Unsal Capkan.

**Project administration:** Davut Unsal Capkan.

**Resources:** Davut Unsal Capkan.

**Software:** Davut Unsal Capkan.

**Supervision:** Davut Unsal Capkan.

**Validation:** Davut Unsal Capkan.

**Visualization:** Davut Unsal Capkan.

**Writing – original draft:** Davut Unsal Capkan.

**Writing – review & editing:** Davut Unsal Capkan.
